# A Cross-sectional Serological Study of Cysticercosis, Schistosomiasis, Toxocariasis and Echinococcosis in HIV-1 Infected People in Beira, Mozambique

**DOI:** 10.1371/journal.pntd.0003121

**Published:** 2014-09-04

**Authors:** Emilia Virginia Noormahomed, Noémia Nhacupe, Carmen Mascaró-Lazcano, Manuel Natane Mauaie, Titos Buene, Carlos Abel Funzamo, Constance Ann Benson

**Affiliations:** 1 Department of Microbiology, Universidade Eduardo Mondlane, Maputo, Mozambique; 2 Department of Medicine, University of California, San Diego, San Diego, California, United States of America; 3 Department of Parasitology, Universidad de Granada, Granada, Spain; 4 Beira Central Hospital, Ministry of Health, Beira, Mozambique; 5 Instituto Nacional de Saúde, Ministry of Health, Maputo, Mozambique; Universidad Nacional Autónoma de México, Mexico

## Abstract

**Background:**

Helminthic infections are highly endemic in Mozambique, due to limited access to healthcare and resources for disease prevention. Data on the subclinical prevalence of these diseases are scarce due to the fact that an immunological and imaging diagnosis is not often available in endemic areas. We conducted a cross-sectional study on HIV1^+^ patients from Beira city in order to determine the seroprevalence of cysticercosis, schistosomiasis, toxocariasis and echinoccocosis and its possible interaction with HIV infection.

**Methodology/Principal Findings:**

Patients (601) were voluntarily recruited at the Ponta Gea Health Center and their demographic and clinical data were recorded (including CD4^+^ cell count and antiretroviral regimen). Mean age was 39.7 years, 378 (62.9%) were women and 223 (37.1%) were men. Four hundred seventy-five (475) patients (79%) were already on highly active antiretroviral therapy (HAART), and 90 started therapy after being enrolled in the study. For serological testing we used a Multiplex Western Blot IgG from LDBIO Diagnostics. The overall seroprevalence was 10.2% for cysticercosis, 23% for schistosomiasis, 7.3% for toxocariasis and 17.3% for echinococcosis.

**Conclusions/Significance:**

Neither age nor the CD4^+^ count were significantly associated with the seroprevalence of the helminths studied. However, patients with CD4^+^ between 200–500/µl had a higher seroprevalence to all helminths than those with less than 200/µl cells/and those with more than 500 cells/µl. Female gender was significantly associated with cysticercosis and schistosomiasis, and being in HAART with toxocariasis. Headache was significantly associated with cysticercosis and toxocariasis. There was no association between epilepsy and seropositivity to any of the parasites. The study concluded that a clear understanding of the prevalence and manifestations of these coinfections, how best to diagnose subclinical cases, and how to manage diseases with concomitant antiretroviral therapy is needed.

## Author Summary

In Mozambique many parasitic diseases persist as a result of low living standards and environmental contamination from human and animal fecal waste. Parasites undermine the health of Mozambique's poorest inhabitants and will be difficult to eradicate without a considerable improvement in the sanitary conditions. Many helminthic infections are undiagnosed because of the need to employ immunologic and image analysis methods, both of which are very costly. We want to know the scope of these co-infections, their possible interaction with HIV, and the course of the disease. We have therefore investigated the seroprevalence against four tisular helminth diseases (cysticercosis, schistosomiasis, toxocariasis and echinococcosis) in a group of HIV infected patients, obtaining data previously absent and relevant conclusions that will enable us to continue working in this field and design strategies in order to improve the quality of life of our people.

## Introduction

The study of HIV and helminth coinfection is a topic of great interest in endemic regions because little is known about the synergism that may exist between HIV and tisular helminths.

Important questions remain regarding the increased susceptibility to helminths, HIV replication enhancement, worsening of HIV-associated neurological disorders, and increased incidence and severity of the immune reconstitution inflammatory syndrome (IRIS) following initiation of antiretroviral therapy.

To clarify the interactions that probably exists it is important to determine the seroprevalence of non-intestinal helminths in HIV infected patients. In this study we have selected cysticercosis, schistosomiasis, echinococcosis and toxocariasis.

Cysticercosis is emerging as a serious public health problem in the countries of Eastern and Southern Africa especially in rural subsistence farming communities, where raising cattle is not economically feasible [1]. In such areas pigs may range freely, having direct access to human feces from outdoor facilities, and veterinary inspection of meat does not exist or is inadequate, thus facilitating the continuous transmission of the disease.

The increasing demand for pork meat in urban areas may result in the transport of infected meat from rural communities to large urban populations. Previous studies of abattoir records indicate the presence of porcine cysticercosis in all provinces of Mozambique [2].

Neurocysticercosis (NCC), the most serious complication of the disease, is associated with seizures, headaches, intracranial hypertension, focal neurological disorders, hydrocephalus, encephalitis, and occasionally with psychiatric manifestations and dementia [3].

Previous serological studies in Mozambique showed that 15 to 21% of apparently healthy adults were positive for cysticercosis, while in neuropsychiatric patients seroprevalence was as high as 51%, [2]. In a study conducted in Angónia District, to evaluate the association between epilepsy and NCC, of 2,023 individuals screened with a *T. solium* cysticercosis antigen ELISA, 15% were positive. Of these, 47% had a history of epilepsy. Additionally, 43 (57.3%) out of 75 individuals positive for cysticercus antigens with associated epilepsy, had brain lesions identified as *T. solium* cysts on computed tomography scan (CT scan) [2].

Urogenital schistosomiasis is a risk factor for contracting HIV in both sexes due to its chronic immunomodulatory effects that lead to a more aggressive infection. In women in particular it causes alterations of the genital mucosa (mucosal edema, abrasion, and ulceration), that persist even after treatment with a specific anthelminthic drug [4].

A survey conducted in Mozambique to assess the prevalence of schistosomiasis in school children found infection rates of approximately 47% *S. haematobium* in urine and 1.5% *S. mansoni* in stools [5]. It is well established that anti IgM and IgG ELISA detection shows much higher prevalences than those obtained from microscopic study, while allowing the diagnosis of coinfection by both species [6].

There is no information about the occurrence of human echinoccocosis in Mozambique although it exists in neighboring countries. In a retrospective study in Tanzania, the incidence has been established in 10 cases per 100.000 people per year [7]. In South Africa it is an agent of morbidity with an estimated prevalence of about 137 patients hospitalized per year. A significantly increased mortality was found in patients also infected with HIV and TB [8].

A possible interaction between echinococcosis and HIV has been pointed out since a depressed immune response may lead to an increased susceptibility for both pathogens and also to a more severe hydatid disease [9].

We have not found previous data on the prevalence of human toxocariasis in Mozambique. Whilst this infection is frequently overlooked, its presence can have serious consequences. A systematic revision and a meta-analysis of available data support the evidence of a positive association between seropositivity to *Toxocara spp* and epilepsy. Cognition will be affected during chronic infections with hyperactive behavior in children and severe mental alterations in elderly adults [10]. In experiments on infected mice it was determined that the telencephalon and the cerebellum, (areas involved in memory and coordination), are the preferred places of larvae accumulation. Furthermore, in murine toxocariasis, the neurodegeneration is associated with the emergence of AβPP and phosphorylated tau in the brain [11].

Although factors that induce occasional migration of tisular *Toxocara* larvae to the brain are not well determined, it is obvious that the immune status is likely to be involved.

## Methods

Patients for this study were recruited at Ponta Gea Health Centre (PGHC), located 5 km from Beira Central Hospital (BCH), the second largest referral hospital, and located in the central part of the country. Those receiving care for HIV-1 infection were approached systematically about participation. Prior to enrollment, a nurse or a research doctor explained the aims of the study to potential volunteers and obtained their written consent. The study was approved by the National Bioethics Committee of Mozambique and the Human Research Protections Program of the University of California, San Diego, US.

The sample size was calculated using Statcalc in Epi Info 3.3.2 software. At a power of 90% and a confidence interval level of 95%, a sample size of 601 was obtained. The bivariate and unconditional logistic regression analysis was carried out to analyze the association between the socio demographic and clinical variables and the prevalence of the parasites [12]. Data collection and sampling were taken from February 2011 to June 2012.

Demographic and clinical data including gender, age, CD4^+^ count (<200, 200–500 and >500), and antiretroviral treatment history, were each recorded and additionally, any clinical signs and symptoms such as headache, epilepsy that might be related to CNS infection.

A 5 ml sample of venous blood was taken by venipuncture and blood serum was removed. This was put into 3 aliquots and frozen until transported from PGHC to the Parasitology Laboratory at Faculty of Medicine, Eduardo Mondlane University in Maputo. Here serological testing was carried out to determine the presence of antibodies against *Taenia solium* larva, *Schistosoma spp*, *Echinococcus spp* and *Toxocara* spp, using the multiplex Western Blot IgG kits for parasites serology from LDBIO Diagnostics (www.ldbiodiagnostics.com). Results were interpreted in each case in accordance with the detailed instructions given by the manufacturer.

Patients were divided by sex, age range (18–25, 26–35, 36–45, +45), and whether or not they had been in HAART previous to the sample collection.

## Results

We enrolled a total of 601 HIV infected patients with a mean age of 39.7 years; 378 (62.9%) were women and 223 (37.1%) were men. A total of 475 (79%) patients were already on HAART and 90 started HAART after being registered in the study. In 10 cases it was not known exactly when HAART therapy had been commenced.

A brain CT scan was performed in 48 patients with positive serology for cysticercosis at Beira Central Hospital (BCH) in order to detect any brain lesions or abnormalities. We defined subclinical cysticercosis as a patient with a positive Western Blot assay result but no overt neurologic signs or symptoms. All results were reported to medical personnel caring for the participants.

Western Blot IgG kits indicated that 61 [10.2%; 47(77%) females and 14(23%) males], were positive for cysticercosis 139 [23%; 71(51%) females and 68 (49%) males], were positive for schistosomiasis, 44[7.3%; 25(56.8%) females and 19(43.2%) males], were positive for toxocariasis and 104 [17.3%; 72(69.2%) females and 32(30.8%) males], were positive for echinococcosis, and (Table 1). Seven patients were seropositive for two parasites; five of them for both echinococcosis and schistosomiasis one for schistosomiasis and cysticercosis, and the other one for cysticercosis and echinococcosis. No patients were seropositive for three or more parasites.

**Table 1:** Seroprevalence against *Cysticercus*, *Schistosoma*, *Toxocara* and *Echinococcus* antigens based on a western blot IgG kit, in HIV^+^ patients from Beira, Mozambique.

		*Cysticercus* N(%)	*p*	*Schistosoma* N(%)	*p*	*Toxocara* N(%)	*p*	*Echinococcus* N(%)	*p*
Sex	Fem	47(77.0%)	0.007*	71(51.1%)	0.0005*	25(56.8%)	0.2	72(69.2%)	0.07
	Male	14(23.0%)		68(48.9%)		19(43.2%)		32(30.8%)	
	Total	61(100%)		139(100%)		44(100%)		104(100%)	
Age years	18–25	5(8.6%)		13(8.1%)		2(4.5%)		5(4.9%)	
	26–35	18(31.0%)		44(32.6%)		16(36.4%)		34(33.0%)	
	36–45	21(31.0%)		43(30.4%)		15(34.1%)		34(33.0%)	
	45+	17(29.4%)		39(28.9%)		11(25.0%)		30(29.1%)	
	Total	58(100%)		135(100%)		44(100%)		103(100%)	
HAART	Yes	32(72.7%)	0.2	71(71.7%)	0.04	33(97.1%)	0.0008*	53(67.9%)	0.01
	No	12(27.3%)		28(28.3%		1(2.9%)		25(32.1%)	
	Total	44(100%)		99(100%)		34(100%)		78(100%)	
Epilepsy	Yes	1 (1.7%)	0.6	2 (1.5%)	0.7	1 (2.3%)	0.5	3 (2.9%)	0.2
	No	59(98.3%)		133(97.5%		43(97.7%)		101(97.1%)	
	Total	60(100%)		135(100%)		44(100%)		104(100%)	

*statistically significant at 95% C.I.

Following analysis of disease prevalence by sex all were found to be higher in women, with gender being significantly associated with cysticercosis (*p* = 0.0145) and with schistosomiasis (*p* = 0.0006) (Table 2).

**Table 2:** Unconditional logistic regression analysis of seroprevalence of *Cysticercus*, *Schistosoma*, *Toxocara* and *Echinococcus* related with CD4^+^ count, age, sex and being or not in HAART.

	*Cysticercus*				*Schistosoma*				*Toxocara*				*Echinococcus*			
	O.Ratio	95% C.I		*p*-value	O.Ratio	95% C.I		*p*-value	O.Ratio	95% C.I		*p*-value	O.Ratio	95% C.I		*p*-value
CD4+	1.0003	0.9992	1.0013	0.6438	1.0003	0.9995	1.0011	0.5092	1.0000	0.9987	1.0013	0.9765	0.9998	0.9989	1.0007	0.6804
Age	1.0052	0.9797	1.0315	0.6902	0.9889	0.9708	1.0074	0.2383	0.9773	0.9484	1.0070	0.1331	1.0072	0.9872	1.0276	0.4835
Sex (M/F)	0.4356	0.2237	0.8484	0.0145*	2.0595	1.3640	3.1097	0.0006*	1.5392	0.7910	2.9948	0.2042	0.6553	0.4066	1.0562	0.0827
HAART (Y/N)	0.5703	0.3057	1.0638	0.0775	0.6746	0.4263	1.0675	0.0928	14.5992	1.9708	108.1471	0.0087*	0.6201	0.3752	1.0247	0.0622

*statistically significant at 95% C.I.

Being in HAART was significantly associated with toxocariasis (*p* = 0.0087). Although seroprevalence to all helminths was more than three times higher in the age groups above 26 years this was not statistically significant, CD4+ count was also not significantly associated with any parasites (Table 2).

Of the patients studied, 9 had a history of epilepsy; among them one was seropositive for cysticercosis, two for schistosomiasis, one for toxocariasis and three for echinococcosis. However, there was no association between epilepsy and seropositivity to any of the parasites (Table 1).

When stratified according to the CD4^+^ cell count, we noted that patients with CD4^+^ cell counts between 200–500 cells/µl had an overall helminthic seroprevalence rate that was 1.6 to 2.5 fold higher than patients with a CD4^+^ count less than 200 cells/µl. Among the same group with CD4^+^ cell count between 200–500 cells/µl there were 1,8 to 2,8 times more seropositive cases than among patients with CD4^+^ cell counts greater than 500 cells/µl. However these differences were not statistically significant (Table 3).

**Table 3:** Seroprevalence of *Cysticercus*, *Schistosoma*, *Toxocara* and *Echinococcus* stratified according to CD4 cell count and been or not on HAART and seroprevalence according to having or not seizures or headache.

		*Cysticercus* N(%)	*p*	*Schistosoma* N(%)	*p*	*Toxocara* N(%)	*p*	*Echinococcus* N(%)	*p*
CD4 ≤200/µL	On HAART	12 (38.9%)	0.4	30 (37.5%)	0.3	10 (29.4%)	0.09	23 (39.0%)	0.4
	Not HAART	5 (35.7%)	0.4	10 (40.0%)	0.4	0 (0%)	0.6	3(14.3%)	0.008*
200–500/µL	On HAART	19 (61.3%)		50 (62.5%)		24 (70.6%)		36 (61.0%)	
	Not HAART	9 (75.0%)		15(53.6%)		1 (100%)		18(72.0%)	
>500/µL	On HAART	13 (40.6%)	0.17	21 (29.6%)	0.2	9 (27.3%)	0.3	17 (32.1%)	0.4
	Not HAART	3 (35.0%)	0.11	13(46.4%)	0.3	0 (0%)	0.3	7 (28.0%)	0.05
Seizures	Yes	2 (3.4%)	0.1	3 (2.2%)	0.1	0 (0.0%)	0.6	0 (0.0%)	0.3
	No	56 (96.6%)	0.1	134 (97.8%)	0.1	44 (100.0%)	0.6	104 (100.0%)	
Headache	Yes	19(31.1%)	0.02*	25(18.1%)	1.18	5(11.4%)	0.05*	20(19.2%)	0.3
	No	42(68.9%)	0.1	113(81.9%)	1.18	39(88.6%)	0.05	84(80.8%)	0.3

* statistically significant at 95% C.I.

When we analyzed the relationships among CD4^+^ cell count, HAART treatment and seropositivity for helminths, we found that, with the exception of schistosomiasis, in the group of patients with CD4^+^ cell count less than 200 cells/µl, those on HAART were more likely to be seropositive, although this was statistically significant only for echinococcosis 23 (39%) (p = 0.008) (Table 3).

In the group with CD4^+^ cell counts between 200–500 cells/µl, the seroprevalence rate was higher in patients not on HAART for cysticercosis and for echinococcosis.

In the group with CD4^+^ cell counts higher than 500 cells/µl the seroprevalence was higher for those on HAART therapy for all helminths except for schistosomiasis, (Table 3).

Among patients who reported having had headache 19 (31.1%) were seropositive for cysticercosis and 5 (11.4%) for toxocariasis. These findings were statistically significant; p = 0.02 and p = 0.05 respectively for cysticercosis and toxocariasis, (Table 3).

We were able to take a CT scan of only 48 patients seropositive to cysticercosis due to the fact that the CT machine broke down several times during the study. None of these patients showed brain abnormalities.

## Discussion

We have used a commercial test for the serodiagnosis due to its economy and reliability. The specific bands within which a result is deemed positive are explained in the manufactures instructions. Nevertheless, it cannot be ruled out that the genetic background of the population, their environmental living conditions or being infected with HIV, has not influenced the results in some way.

Seroprevalence for tissue helminths in HIV infected patients was demonstrated to be high, with schistosomiasis being the most frequent affection (23%), followed by echinococcosis (17.3%), cysticercosis (10.2%) and toxocariasis (7.3%). The data analysis is complex as there are many factors involved but the existence of a high percentage of HIV-helminths coinfections is clear.

We have conducted previous studies on schistosomiasis and cysticercosis in Mozambique [2], [5], but not investigated the prevalence of toxocariasis and echinococcosis, so one limitation of our study is the lack of seroprevalence data of echinococcosis and toxocariasis in HIV negative population. Consequently these results must be interpreted with care as it is difficult to obtain the right conclusions. We did not search for hydatid cysts in the seropositive patients due to financial limitations.

These helminthiasis are chronic conditions that, in the absence of treatment, remain in the body for prolonged periods, although by themselves are not associated with immunosuppression because they are not opportunistic pathogens.

As we have indicated one of the most common presentations of AIDS in our environment is uncontrollable diarrhea. Patients attending health services for this reason are routinely prescribed albendazol, mebendazol or cotrimoxazol. We do not know whether these drugs may have in some way influenced the results of this study.

CD4^+^ cell count was not significantly associated with any parasite. However, the global seroprevalence was higher (although not significant) in patients between 200–500 CD4^+^/µl than in those with count less than 200 or greater than 500 (Table 3). A possible explanation could be that although all groups have the same susceptibility to the infections patients with CD4^+^ count <200 are highly immunocompromised having lost the ability to produce antibodies, or producing them at minimum levels undetectable with our assay. It is worth considering that since these are chronic conditions and non-opportunistic parasites some of these patients might have been already infected before contracting the HIV. A population study among the non-HIV infected people from the same region would provide data on the prevalence of these conditions at different ages.

In the case of patients with CD4^+^ >500 cells the explanation could be because their immune system is not yet compromised thus they have more ability to clear out the parasites before they become established in the body.

The high seroprevalence of schistosomiasis is not a surprise, as it is a common condition in the country [5]. Its significant association with female gender probably contributes further to the risk factors that lead to the higher HIV prevalence in females, due to the alterations in the integrity of the female genital mucosa that favor the transmission of the HIV virus in addition to the immunomodulatory effects of chronic schistosomiasis. The WHO recommended that regular treatment of children with praziquantel needs to be extended to adults and prioritized in national programs as a possible means of further preventing HIV infections in sub-Saharan countries [4].

In a previous serological study of echinococcosis using ELISA and AgB5 a total of 269 sera from apparently healthy young people from a suburban area of Maputo city were negative [13]. In the present study echinococcosis seroprevalence was surprisingly high, 17.3% (104 patients). In contrast to the high seroprevalence we found in this study, there are no reported cases of human hydatidosis in the country. The only one reported was post mortem, a patient who had been working in South Africa and it was therefore impossible to establish the source of his infection.

We did not search for hydatic cysts in liver or lung using ultrasonography or CT scan due to the financial limitation. In neighboring countries like South Africa, Tanzania and Zimbabwe surgical interventions in patients with hydatid cysts are performed. Cross-reactions with cysticercosis can be discarded as only one of the sera tested positive for both helminthiasis.

Recent studies have demonstrated that diversity of *Echinococcus* species and strains is greater in Africa than on any other continent. Variability by molecular epidemiological research has been studied in South Africa where *E. granulosus* sensu stricto G1–G3, *E. canadiensis* G7 and *E. ortleppi* G5 are all known to exist. Their global seroprevalence is 17.0% with the Eastern Cape Province showing an even higher rate of 30.4% [14]. Much research is needed in our country in order to clarify which species and strains exist, their infectivity for humans, their clinical manifestations and the hosts implicated in their life cycles in order to establish preventive measures. It has been suggested that HIV and TB induced immune modulation seems to affect the clinical course of echinococcosis [15]. In fact we have found in this study a strong association with HAART in patients with CD4^+^ cell count less than 200/µl and seropositivity to echinococcosis. However, is difficult to interpret this result because of the lack of data on this disease in our country. It is well known that different *Echinococcus* strains differ in their preferential location (liver, lungs, etc.) both in humans and in other hosts and can also be incompletely developed in some giving rise to infertile cysts (acefalocystic).

In African countries only now are we beginning to study this diversity, which, it is hoped will shed light on human hydatidosis [15], [16]. A recent epidemiological study in Kenya looking at the prevalence of hydatidosis in cattle, sheep and goats shows a significant increase compared with data about three decades ago [17]. Even the introduction of livestock from other countries after the civil war could be a factor involved in echinococcosis in a country like Mozambique, with such an abundant canine population in its rural environment.

Cysticercosis seroprevalence did not surprise us because previous studies had already established the problem when identifying what our priorities should be in researching this area [2].

Unfortunately cysticercosis continues to be ignored, underdiagnosed and neglected. As Mozambique is still lacking enough epidemiological data about human taeniasis and cysticercosis, it is impossible to draw firm conclusions on its prevalence and geographical distribution. Its impact on human health in both HIV positive and negative populations in our country is still unknown.

If we take into account that patients who are immunocompromised have partially lost the ability to produce antibodies, the seroprevalence to those helminths is probably underestimated. Previous studies in Mozambique showed that in apparently healthy people cysticercosis seroprevalences were much higher than we have determined in this study [2].

Magnetic Resonance Imaging (MRI) is the ideal technique to detect neurocysticercosis lesions, but we have no access to this equipment. Image brain studies are scarce in Mozambique and mainly accomplished by means of CT scanning, a lesser expensive technique. Although only 48 patients underwent CT, we did not find any with brain lesions. This could be due to the small sample size, or related to the genetic variants of *T. solium* that circulates in our setting. It has been documented that there is a Latino/African genetic variant and an Asian genetic variant of *T. solium* each correlated to a different clinical presentation. Serological analysis of cyst fluid of *T. solium* cysticerci obtained in China, Indonesia, Mozambique and Ecuador indicates geographical differences in their banding patterns, since there are some varieties of manifestation of neurocysticercosis with or without subcutaneous cysticercosis and possible differences in the pathology of cysticercosis worldwide [18].

There is growing evidence that long-standing headache is one of the frequent symptoms after epilepsy in patients with NCC. Our study has shown that people with positive serology had significantly more headaches than those with negative serology and this should be taken into account in the future for the diagnosis of cysticercosis.

The immunomodulatory mechanisms induced by cysticercosis showed themselves to be very complex [19]. These finding seems logical, being a metacestode that can not only occupy different locations but also be found in various different stages of development, degeneration or calcification.

Genetic variances of the host might explain the susceptibility of people with NCC to develop symptomatic diseases, and polymorphisms of toll-like receptor 4 (TLR 4) have been implicated in this process [20]. In Mozambique we do not know what genetic variants circulate and further studies should be directed at identifying the variants we have as we were exposed to European migration as well as Asian migration in the past.

Toxocariasis is a zoonotic cosmopolitan infection more prevalent in tropical areas with poor hygiene, where soil is highly contaminated with *Toxocara spp* eggs from dogs and cats and can survive for a long time in humid soil.

There are very few studies on the prevalence of *Toxocara* ocular and visceral *larva migrans* in sub-Saharan countries. Seroprevalence is high (44.6%) among Swaziland children living in rural slums [21], in Cameroon a 36,3% of young people under 20 years were seropositive, and from 14 people with epilepsy 5 were seropositive to *Toxocara* antigens [22].

We do not have any comparative data on HIV-noninfected population from our country nor any other work on the seroprevalence of toxocariasis in HIV^+^ patients, but we have found a significant association with being in HAART and also with headache.

The low frequency of sera reacting to more than one parasite antigen could be explained by the fact that not all have the same path of transmission and to the impaired immune system that underestimates the seroprevalence due to the lower antibodies titles.

Compounding these problems is the fact that even before HIV has been diagnosed, these patients are routinely given albendazole, mebendazole or other medication such as cotrimoxazole to treat complaints such as diarrhea, which is one the most common presentation of AIDS in our settings. Even though there are given albendazole or mebendazol at a certain point for their disease it does not explain at all this low rate of multireactive sera because the dose is insufficient to kill the larvae of *T. solium* and *Echinococcus*.

As a general result of this study, we conclude that in our wormy world HIV^+^ patients showed a high seroprevalence versus the four non-intestinal helminths surveyed. These chronic conditions are all derived from environmental contamination with human and animal feces. Much remains to be learned about its synergism with HIV and other associated pathogens, being important to approach in our country the study of echinococcosis and toxocariasis, possibly two new still uninvestigated zoonoses.

## Supporting Information

Supporting Information S1 STARD checklist.
(DOC)

Supporting Information S2 A brief summary diagram.
(DOCX)
