# Supplementary material for: A Cross-sectional Serological Study of Cysticercosis, Schistosomiasis, Toxocariasis and Echinococcosis in HIV-1 Infected People in Beira, Mozambique
Source: PLoS Negl Trop Dis. 2014 Sep 4;8(9):e3121. doi: 10.1371/journal.pntd.0003121 (PMC4154675; doi:10.1371/journal.pntd.0003121)
Supplement: Supporting Information S2 — A brief summary diagram. (DOCX) [file pntd.0003121.s002.docx]

A Cross-sectional Serological Study of Cysticercosis, Schistosomiasis, Toxocariasis and Echinococcosis in HIV-1 Infected People in Beira, Mozambique
